# Interclonal Variation in Heavy Metal Accumulation Among Poplar and Willow Clones: Implications for Phytoremediation of Contaminated Landfill Soils

**DOI:** 10.3390/plants14040567

**Published:** 2025-02-13

**Authors:** Branislav Kovačević, Marina Milović, Lazar Kesić, Leopold Poljaković Pajnik, Saša Pekeč, Dragica Stanković, Saša Orlović

**Affiliations:** 1 Institute of Lowland Forestry and Environment, University of Novi Sad, Antona Čehova 13d, 21102 Novi Sad, Serbia; katanicm@uns.ac.rs (M.M.); kesic.lazar@uns.ac.rs (L.K.); leopoldpp@uns.ac.rs (L.P.P.); pekecs@uns.ac.rs (S.P.); sasao@uns.ac.rs (S.O.); 2 Institute for Multidisciplinary Research, University of Belgrade, Kneza Višeslava 1, 11030 Belgrade, Serbia; dstankovic@imsi.bg.ac.rs

**Keywords:** phytoextraction, landfill, heavy metals, *Populus* sp., *Salix* sp.

## Abstract

In this study, five poplar clones (*Populus deltoides* cl. PE19/66, cl. S1-8, cl. 135/81, and *Populus × euramericana* cl. I-214, cl. Pannonia) and two white willow clones (*Salix alba* cl. 380, cl. 107/65-9) were tested in pot trials. The aim was to evaluate their potential for phytoextraction of nine heavy metals (Cd, Cr, Cu, Fe, Mn, Ni, Pb, and Zn) in three substrates, two based on soil from landfills near Belgrade and Novi Sad, and one control treatment based on nursery soil. The shoot content of all analyzed heavy metals was the highest in the BG substrate with the highest content of heavy metals and the lowest in the control substrate. White willow clone 107/65-9 achieved the highest accumulation of Cd, Cr, Fe, Ni and Pb and along with another willow clone 380 is found to act as generalists. Poplar clones performed more as specialists: I-214 and Pannonia for copper, PE 19/66 for manganese and S1-8 for nickel and zinc. Considerable differences among examined clones in heavy metal accumulation and reaction to substrates should be taken into consideration in further pot and field trials as well as in phytoremediation projects on landfills.

## 1. Introduction

Accumulation of heavy metals in the environment poses a considerable threat for all living beings. Although some heavy metals are essential for growth and development of plants (Cu, Zn, Mn, Fe, Ni, Co, Mo), in high concentrations could be harmful too [1,2]. On the other hand, heavy metals Cd, Pb, Hg, As and Cr are defined as environmental pollutants because they have detrimental effects on environment even if present at very low concentrations [3]). Since heavy metals cannot be degraded, they accumulate in the environment and afterwards contaminate the food chain. This contamination represents a long-term threat to the health of humans and environment as well [4,5].

Landfills produce gas and leachate whose emissions are considerably affected by biological processes occurring in them [6]. Rain has a major influence in the formation of leachates because precipitation filters through the deposited waste and binds to waste constituents [7]. Leachates contain four main components: nutrients (namely nitrogen), volatile organic compounds, toxic organic compounds, and heavy metals. A great deal of these substances occurring in landfill leachates is hazardous and toxic to the environment and human health. Moreover, chemicals can be accumulated in organisms and proceed to the food chain, eventually reaching humans [6]. Thus, landfill treatment is necessary to avoid contamination of ground- and surface waters.

There are a variety of remediation approaches developed to restore heavy metal-contaminated sites, but these measures are mainly based on mechanical or physico-chemical techniques [4,5]. An alternative treatment is to use species and hybrids of the genera *Populus* and *Salix* in remediation of the landfills [8,9]. Phytoremediation is a technology which uses plants and associated soil microorganisms to reduce the concentration or toxic effects of contaminants in different environments. It is a relatively new, efficient, novel, cost-effective, and eco-friendly technology that is solar-driven [4].

Plants with extensive root systems can absorb and sequester heavy metals and other contaminants or reduce their bioavailability in the soil. This process not only cleanses the soil but also helps improve its fertility [5]. Moreover, modern research suggests that phytoremediation should be seen beyond just contaminant removal. It provides also multiple ecosystem services, such as improving soil structure, increasing biodiversity, and enhancing carbon sequestration [10]. This holistic approach contributes to sustainable land management and ecosystem restoration.

Phytoextraction is the most important phytoremediation technique used for reclamation of heavy metals and metalloids from the polluted soil [4]. It is based on the use of plants to take up contaminants from soil or water; it translocate them and accumulate in aboveground plant organs [4,5]. Phytoextraction is a permanent solution for the removal of heavy metals from polluted soil and, therefore, is more suitable for commercial applications. The efficiency of phytoextraction relies on plant selection, plant performance, heavy metal bioavailability, soil, and rhizosphere properties [4]. Appropriate selection of the plant species is vital for effective phytoextraction. According to [4], the plant species for phytoextraction should possess high tolerance to the toxic effects of heavy metals, high extraction ability with accumulation of high levels of heavy metals in the aboveground parts of the plant, fast growth with high biomass production, abundant shoots and extensive root system, and mechanism of good adaptation.

Poplars and willows have been utilized in a variety of phytoremediation projects [11,12,13,14,15,16]. These plant species are ideal for remediation due to their rapid growth, large biomass production, efficient photosynthesis, extensive root systems, intensive transpiration, easy and inexpensive propagation from hardwood cuttings, and growth on marginal lands [17,18]. However, the potential for phytoextraction of heavy metals largely differs between species and genotypes, as indicated by numerous studies [14,15,19,20,21]. It could be one of the reasons why [22] found, in meta-analysis a study, that results of the accumulation patterns of metals in *Populus* spp. were contradictory in the earlier phytoremediation studies. Namely, they revealed that accumulation of Cd, Cr, and Zn was significant in all plant parts (root, stem and leaf), while for Cu and Pb it was significantly higher in the root and leaf, but not in the stem. Moreover, the accumulation in all plant parts was moderate for Ni and limited for Mn. In poplars of different age groups, [23] found considerable translocation of Cd, Zn and Mn into the leaves, but not for Pb and Ni. Also, [24] aimed to determine the phytoextraction potential of four poplar hybrids under greenhouse conditions and demonstrated higher Zn accumulation in leaves, compared to Cd, Cr, and Cu. In a 4-year field study, [25] evaluated the phytoextraction efficiency of *Populus deltoides* CL. ‘Xianglin 90’ grown on mine tailings co-polluted by Cd, Cu, Cr, Ni, Pb, and Zn, revealing that the most extraordinary bioaccumulation and root–shoot translocation patterns occurred for Cd and Zn. These findings supported the importance of the heavy metal- and growth condition-specific application of poplars in phytoremediation processes.

According to [19], willow species *Salix alba* and *S. viminalis* are good accumulators of Ni, Cu and Cd, and the best translocation to the leaves was obtained for Cd. Furthermore, [26] noted that willow clones from the unpolluted area had higher accumulations of Cd, Cu, and Zn in their roots and a lower transport of heavy metals to the shoots than willow clones from the polluted area. Also, [27] found that the ability of the willow clones to extract and translocate Cd, Ni, and Pb differed depending on the quantity of metal content in the nutrient solution and on the willow genotype. On the other hand, [20] stated that contents of determined metals in twelve analyzed species, varieties, and willow genotypes varied both in the range of metal concentrations and also in the type of plants. According to [28], the poplars produced a higher amount of biomass than willows, but willow clones accumulated higher amount of Cd, Zn, and Cu in their biomass. Also, [9] noted that the poplar clones *P. deltoides* cl. S 1-8 and PE 4/68 exhibited superior physiological performance across multiple parameters under suboptimal soil conditions present at landfill sites.

In order to identify and select superior-performing clones for specific remediation efforts phyto-recurrent selection can be used [8]. Specifically, this method involves the evaluation, identification, and selection of suitable clones using multiple testing cycles. The number of clones tested in each cycle decreases as the complexity of the data increases [29]. Currently, there is a growing need to develop drought-resistant and contamination-tolerant clones and cultivars, which are not only successful at phytoremediation but are also able to mitigate climate change by sequestering excess carbon dioxide through their large biomass [9].

The aim of this study was to assess differences between studied poplar and willow genotypes in their potential for phytoextraction of different heavy metals, using pot trials with substrates based on the soil from two landfills. Furthermore, the final objective was to select the most suitable genotypes for the phytoremediation of landfill soils contaminated with heavy metals, both in terms of overall effectiveness and for each specific heavy metal that was analyzed.

## 2. Results

### 2.1. Substrate Characteristics

As shown in Table 1, soil textures of soil from experimental estate (control), the Belgrade (BG) and Novi Sad (NS) landfills were sand, clay loam, and sandy loam, respectively. Soil from Belgrade landfill had the lowest content of CaCO_3_ but the highest content of N, P_2_O_5_ and K_2_O. The measured pH values of all analyzed soils were alkaline and soil from the Novi Sad landfill and had the highest pH. The control soil had the lowest content of humus, N, P_2_O_5_ and K_2_O (Table 1).

In the soil from the Belgrade landfill, concentrations of Cd, Cu and Ni were above the maximum limit value (MLV) [30] while in the soil from the Novi Sad landfill, only the concentration of Cd was above MLV. None of the analyzed heavy metals had soil content higher than the remediation value (RV) (Table 2).

### 2.2. Analysis of Plant Material

Among the analyzed heavy and toxic metals, arsenic and mercury were detected in small quantities in the BG and NS substrates, respectively, while barium was found in all three substrates in much larger quantities (Table 2). However, none of these three metals were found in detectable quantities in the shoot tissues of the studied clones and, therefore, these metals were excluded from further analysis.

The analysis of variance revealed statistically significant effect of all controlled sources of variation on examined heavy metals, except for the Interaction Substrate × Clone effect on shoot cupper content (Table 3).

According to Tukey’s HSD test, the shoot contents of all examined heavy metals were the highest on the BG substrate. The shoot contents of iron, manganese, and lead were significantly higher on the NS than on the control substrate, while the shoot contents of cadmium, chrome, and nickel on these two substrates were not statistically different. The shoot content of zinc was even significantly higher on the control substrate than on the NS substrate.

Tukey’s test confirmed differences between clones in accumulation of heavy metals in shoots. Willow clones, especially 107/65-9, achieved significantly higher accumulation of cadmium, chrome, iron, and lead compared to all or most of the poplar clones. Euroamerican poplar clones I-214 and Pannonia had the highest content of copper, and this is the only case when these Euroamerican poplar clones differed considerably from eastern cottonwood clones. The Pannonia clone and eastern cottonwood clone PE19/66 had the highest content of manganese, while eastern cottonwood clones 135/81 and S1-8 achieved the highest accumulation of nickel (along with white willow clone 107/65-9) and zinc (along with white willow clone 380) (Table 4).

There were differences in the reaction of the analyzed clones on Belgrade and Novi Sad landfill substrates, considering accumulation of the examined heavy metals, especially between willow and poplar clones. There were no significant differences between willow and poplar clones in accumulation of copper, manganese, nickel, and zinc within the same substrate treatment. However, it has been found that willow clones achieved significantly higher content of cadmium, chrome, iron, and lead on the BG substrate compared to the NS substrate.

The difference in the content of heavy metals in the examined clones on the BG substrate and in the control treatment was, in most cases, significant in the examined willow clones, except for copper and zinc. However, this was not the case with the NS substrate, where only willow clone 107/65-9 achieved significantly higher accumulation of iron and lead compared with the control treatment. Examined poplar clones achieved considerable accumulation in few cases on the BG substrate. Clones S1-8 and Pannonia achieved higher content of cadmium, clone PE19/66 achieved higher content of nickel, and most of poplar clones (except S1-8) achieved higher accumulation on the BG substrate than in the control treatment. None of the poplar clones achieved significantly higher content of any examined heavy metal on the NS substrate than in the control treatment (except for lead content in S1-8).

### 2.3. Principal Component Analysis

**Figure 1:**
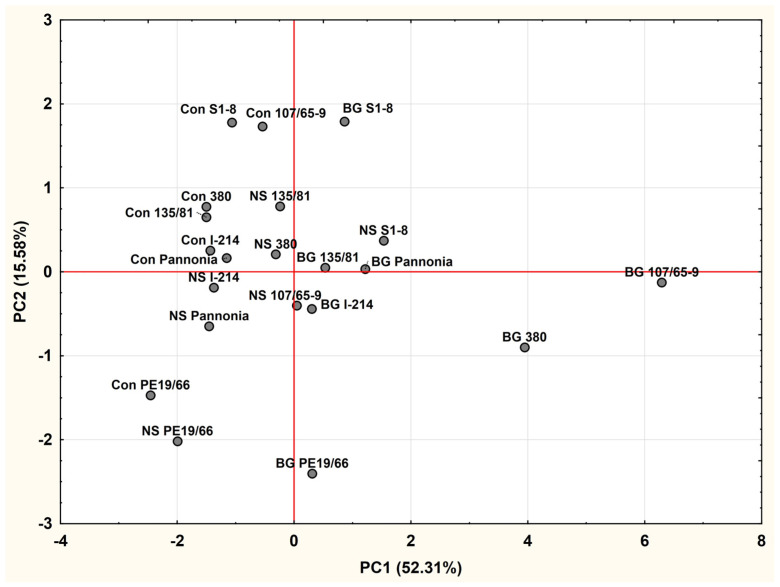
Relationship between Substrate × Clone interaction treatments according to factor scores of the first two principal components (BG stands for Belgrade landfill substrate, NS for Novi Sad landfill substrate, and Con for the control substrate, based on soil from ILFE estate in Kać). Clones 107/65-9 and 380 are willow, and the rest are poplar clones.

The relationship between Substrate × Clone interaction treatments was also examined by a 2D plot based on the first two principal components that explain 67.89% of the total variance. According to these results, treatments on the control substrate are all on the negative side of the first principal component. Also, most of them, except for the clone PE19/66 in the control treatment, are on the positive side of the second principal component. Treatments on NS landfill substrate resemble similarities with the control treatment because almost all of them are on the negative side of the first principal component, while the treatments on BG landfill substrate are on its positive side. Also, it seems that most of clones on NS and BG landfill substrates formed one relatively uniform group with clones on the control substrate, from which willow clones 107/65-9 and 380 on the BG substrate are separated by the first principal component, while treatments of the eastern cottonwood clone PE19/66 are separated by the second principal component (Figure 1).

The analysis of the relationship between the examined parameters was based on the loadings of the original variables with the first four rotated principal components that explained 91.8% of the total variance. In that sense, four groups of parameters were formed according to principal components with which they had their highest loadings. The first group consisted of contents of cadmium, chromium, iron, and lead; in the second group, there were contents of nickel and zinc; in the third group, there was manganese; and in the fourth group, there was copper (Table 5).

### 2.4. Bioavailability

The bioavailability factor was presented for the analyzed clones on each examined substrate. Since cadmium was not detected in the control substrate, the bioavailability of Cd for the examined clones on the control substrate was not calculated. In total, it is evident that the bioavailability factor was, in the most of clones, the highest on the control substrate (except for chromium and manganese), and the lowest on the BG substrate (except for iron), with rare cases when the clones reacted differently from this trend (Table 6). On the BG landfill substrate, where the clones achieved the lowest BFs, the willow clone 107/65-9 had the highest BF for the majority of the examined heavy metals, such as cadmium, chromium, iron, nickel, and lead, while for all other heavy metals, the bioavailability index was higher than the average value for all clones. This clone achieved even higher BF for cadmium, chromium, and zinc on the BG substrate than for the same heavy metals on the NS substrate. Some of the poplar clones achieved interesting BF, such as I-214 on the BG substrate, which achieved the highest BF for copper, PE19/66, which achieved the highest BF for iron on unfavorable the BG substrate, while S1-8 on the NS substrate achieved the highest BF for iron of all treatments, and S1-8 on the BG substrate, which achieved the highest BF for zinc.

## 3. Discussion

Considering the need and benefits of phytoremediation technology utilization, the development of the methodology for the selection of species and genotypes that would efficiently remove hazardous substances is of utter importance. In our study, the soils from two landfills, where phyto-buffers were intended to be established, were tested. Although soils from these landfills were found not to exhibit significant levels of pollution, there is still a danger that heavy metals could reach belowground waters and enter nutritional chains, ultimately endangering the health of the human population. In this study, we evaluated the possibility of the utilization of pot trials in the selection of the best willow and poplar genotypes according to their efficiency in phytoextraction of several heavy metals. To achieve this objective, phyto-recurrent selection, proposed by [8,29], was used. By this selection process, the greater number of potential clone candidates were evaluated to define generalist clones that would be appropriate to remediate a broad range of contaminants or specialists that would match some specific pollutant(s). Differences in the reaction of genotypes to environmental conditions (G × E interactions) are the basis for the characterization of genotypes as generalists or specialists [31,32]. In that sense, given the same mean fitness, ecological specialists would have narrower niche than ecological generalists and higher environmental variance in fitness across the range of patches considered [32]. In this study, an attempt was made to evaluate the ability of examined poplar and willow clones for phytoextraction of heavy metals in landfill soil-based substrates in order to classify them as specialists or generalists and define their use in phytoremediation projects.

Grouping of heavy metal content traits, based on the factor loadings with the first four principal components, showed close relationship between the accumulations of the examined heavy metals of the first group. It seems that the examined clones reacted in a similar way in terms of cadmium, chromium, iron, and lead accumulation in shoots, suggesting similar mechanisms of uptake and accumulation for these heavy metals [2]. Thus, the same clones could be proposed for use in phytoextraction projects on areas contaminated with some of these heavy metals or all of them. Our finding is of special importance, particularly because Cd, Cr and Pb are significant environmental pollutants. Furthermore, in most cases, white willow clones 107/65-9 and 380 achieved higher content of the heavy metals mentioned than the majority of poplar clones, which supports the findings of [33] on agricultural land. However, [14] found no significant differences in physiological responses of poplar (*Populus deltoides* cl. “Bora”) and willow (*Salix viminalis* L. cl. “SV068”) on soil substrate amended with heavy metals (Cr, Cu, Zn, Ni, Cd, and Pb) from contaminated dredged river sediments.

According to the results obtained in our study, the bioavailability factor in total was the highest in the control substrate (except for chromium) and the lowest in the BG substrate (except for iron). It seems that higher concentrations of heavy metals in the BG substrate had an inhibitory effect on the phytoextraction of most of the analyzed heavy metals, except in the case of the white willow clone 107/65-9. Growing four poplar and two willow clones in pot experiments under greenhouse conditions, [34] found a significant decrease in gas exchange parameters and water use efficiency with the increase in Pb, Cd, and Ni substrate content. In a pot experiment with three eastern cottonwood clones (*P. deltoides* Bartram ex Marshall cl. PE 19/66; cl. PE 4/68; cl. S 1-8) and three willow clones (*S. alba* L. cl. 380; cl. 107/65/9; cl. 79/64/2), grown also on landfill soil substrate, [9] found better physiological responses of poplar clones. It seems that higher accumulation of heavy metals of the first PCA group in white willow clones observed in our study could lead to phytotoxicity, causing poorer physiological parameters in willow clones than in the study of [9]. Also, [35] found significant differences between poplar clones Pannonia B229 and PE 19/66 in oxidative stress parameters, and their responses to soil contamination with heavy metals (Ni, Cu, and Cd). Similarly, in the study of [28], the poplars produced a higher amount of biomass than willows, but willow clones accumulated higher amount of Cd, Zn, and Cu in their biomass. In our study, we found that toxicity caused by higher accumulation of heavy metals in willow clones could be, at least partially, caused by worse physiological responses of willow clones. Thus, we assume that toxicity, due to high accumulation of heavy metals, should be taken into consideration in areas where the contents of the examined heavy metals considerably exceed levels that were found in the BG substrate, especially in the case of poplar clones. In a pot experiment conducted with fourteen *Salix* clones on lead/zinc and copper mine tailings, [36] found negative effect of high substrate content of heavy metals on survival and biomass accumulation and considerable differences between the examined clones in tolerance and the accumulation of the analyzed heavy metals. However, [36] found no significant differences in concentrations of Cd, Cu, and Zn in the collected stems of willow clones grown on unpolluted and the polluted areas, due to the lower translocation of heavy metals on polluted compared to unpolluted areas. On the soil with high cadmium content, [34] found better tolerance of clone Pannonia compared to clone B-229 regarding the photosynthetic rate, suggesting better phytoextraction capacity of the clone Pannonia. Also, [14] suggested that, in general, differences in soil content of heavy metals preferentially affected growth in poplar clones, while physiological parameters were more influenced in willow clones. Our findings stress the necessity of further research on the potential phytotoxic effect of heavy metals in landfill soil, especially in the field trials, in order to reach the final conclusions.

Classification of zinc, manganese, and copper shoot content into separate PCA groups from those of the first PCA group suggests different reactions of the examined clones on these three heavy metals. Indeed, some poplar clones dominated, particularly in accumulation of the clone S1-8 in Zn, clone PE19/66 in Mn and clones I-214 and Pannonia in Cu. These results align with previous studies, indicating that genotype-specific adaptations, such as variations in antioxidant enzyme activities and metal transport mechanisms, play a crucial role in heavy metal uptake and tolerance in poplar clones [37]. For instance, clone PE19/66 exhibited strong manganese uptake, which is linked to elevated metal-binding proteins and regulated hormone levels (e.g., ABA and IAA), mitigating metal toxicity and enhance growth [37]. Same authors suggested that these hormonal adjustments are crucial in the mitigation of the toxic effects of manganese, thereby promoting better growth and metal accumulation. In our study, accumulation of nickel, which was the highest in the poplar clones 135/81 and S1-8, did not show high factorial loadings, i.e., clear similarities with any of the formed PCA groups. This variability may be due to the distinct physiological and biochemical mechanisms underlying nickel uptake, such as specific transporters or root exudate production, which do not align with the uptake patterns of Zn, Mn, or Cu, as suggested by [38]. Such findings underscore the specialization of these poplar clones for specific metal accumulation, highlighting their potential utility in targeted phytoremediation applications on contaminated sites.

The relationship between interaction treatments Clone × Substrate, presented at the base of the first two principal components, showed that most of the treatments were concentrated in the main group around the origin. There were several outlawyers on the positive side of the first principal component (clones 107/65-9 and 380 on the BG substrate), indicating their superiority in accumulation of the heavy metals of the first group on the BG substrate. High positive values of the second principal component for clones 107/65-9 and S1-8 on the control substrate and S1-8 on the BG substrate, suggesting superiority of eastern cottonwood clone S1-8 in accumulation of zinc and nickel on the control and BG substrates, which acted as specialists, and confirms the generalist role of white willow clone 107/65-9. On the other hand, negative values of the second principal component for treatments of clone PE19/66 on all three substrates correspond with poor accumulation of zinc by PE19/66 on any of the substrates.

The examined species differed in phytoextraction properties. The white willow clones 107/65-9 and 380 showed characteristics of generalists, achieving higher shoot contents of heavy metals and bioavailability factors. On the other hand, some poplar clones showed characteristics of specialists, showing affinity for specific heavy metals in total, such as I-214 and Pannonia for copper, PE19/66 for manganese, and S1-8 and 135/81 for nickel and zinc. Thus, the relatively good results of PE19/66 in Mn accumulation and of S1-8 and 135/81 in Ni accumulation propose these clones to be used specifically on areas contaminated with heavy metals as they have affinity.

The selection of plant species is an important step in the design of phytoremediation projects [21]. Along with considerable biomass accumulation, woody plant species are especially admired for their relatively deep and extensive root system, which is suitable for the phytoextraction and phytostabilization of heavy metals and other contaminants in a much larger volume of soil compared to the roots of herbaceous plants. White willow is well known for its high accumulation of heavy metals, which is especially supported by their rapid growth. Thus, it could be said that high phytoextraction potential of willow clones in this study was expected [19]. According to [19], significant differences in the concentration of heavy metals were recorded between plants grown on contaminated and control media, especially for Ni, Cu, and Cd. However, it should be taken into consideration that, in this pot trial, the plants were provided with enough water for sustainable growth and development. Availability of water may be an issue on landfills. In that sense, poplar plantation could be beneficial in cases where available water is not sufficient for the growth of willow plantations. On flooded soil, [39] recorded good performance of the 15 *Salix* clones studied in the accumulation of Cd, Zn, and Pb, and found differences between these clones in their multimetal remediation capacity.

There was a distinct difference between the examined substrates in their granulometric composition, where the BG substrate was characterized by higher content of clay + silt fraction than the other two substrates. This difference can considerably influence the bioavailability and uptake of heavy metals by plants where particles of clay and silt have a larger specific surface area and adsorption capacity, allowing the accumulation of higher amounts of heavy metals [40,41]. However, clay layers often exhibit a high cation-binding capacity due to their negatively charged surfaces, which can reduce the bioavailability of heavy metals to plants [41,42]. In that sense, the reaction of the examined clones on the analyzed substrates can be of interest in optimizing phytoremediation strategies and selecting plant species and clones suitable for degraded soils contaminated with heavy metals.

Our study showed that the bioavailability of the analyzed heavy metals, in general, declined with the increase in clay, silt, and heavy metal soil content, with two exceptions: the BF of Cr was not the highest on the control substrate, and the BF of Fe was not the highest on the BG substrate. However, in the study of [22], there was no significant relationship between the soil content of heavy metal and its accumulation in plant tissue. One of the causes of such findings could be the different reaction of genotypes used in their study, as well as the fact that they only analyzed data for poplar species. The landfills are expected to be contaminated with numerous heavy metals, and their phytoremediation would require the use of genotypes that would be able to accumulate and tolerate multiple heavy metals, i.e., generalist clones. The highest contents of the examined heavy metals were found in the BG substrate, especially the levels of iron, manganese, nickel, lead, and zinc. Clones that react to such a high content of heavy metals in substrate with higher accumulation could be selected for use in highly contaminated areas.

Despite the lower expected bioavailability on substrate with higher clay and silt content, white willow clones 107/65-9 and 380 achieved higher accumulation of cadmium, chromium, iron, manganese, nickel, and lead on the BG substrate than on the control and NS substrates. Also, clone 107/65-9 presented dominant performance, achieving the highest BF for the majority of the examined heavy metals, while for the others, the BF values were above the total mean value for all clones. White willow clone 380 also performed well and achieved higher BF for the majority of the examined heavy metals on all substrates compared to poplar clones. These results suggest that the examined white willow clones could be considered as a generalist and be proposed for the establishment of phytoremediation plantations on the areas contaminated with numerous heavy metals that provide appropriate conditions for the growth of willows, especially sufficient water availability. Our results are particularly important, because previous studies showed relatively poor translocation of heavy metals into the shoots [26,43] and considerable differences between willow clones in heavy metal accumulation [36].

On the other hand, only a few poplar clones achieved higher content of specific heavy metals on the highly contaminated BG substrate compared to the less contaminated NS substrate. Clones Pannonia and S1-8 showed high affinity for cadmium, 135/81, I-214, Pannonia, and PE19/66 for iron and PE19/66 for nickel. Also, some of them achieved higher BF on the highly contaminated BG substrate, but specifically for a single heavy metal, such as I-214 for copper, Pannonia and PE19/66 for iron, and S1-8 for zinc, while others achieved BF below or around the mean value for all clones on BG substrate. These mentioned clones show the characteristics of specialists and could be useful for phytoextraction in areas contaminated with a specific heavy metal according to which the clone would be selected. However, if some sites suitable for poplar growth are contaminated with several heavy metals, like in landfills, in lack of generalists, the dense multiclonal short rotation coppice plantations would be proposed, established with a group of clones that would be able, as a group, to mitigate contamination of several heavy metals.

The better performance of plants on the control substrate than on the NS and BG substrates was confirmed by the results of BF, which described plants’ potential for phytoextraction. According to these results, the highest BF in total was achieved on the control, and the lowest on the BG substrate. It seems that it is challenging for a plant to respond to elevated heavy metal content in the soil with its increased accumulation. In that sense, bioavailability could be used as a reliable parameter for the selection of poplar and willow genotypes with high potential for phytoextraction at sites with relatively high heavy metal content. The clones that are able to achieve higher BF, especially on the substrate with relatively high heavy metal content, could be suitable for use in phytoremediation projects on heavy metal-contaminated areas.

## 4. Materials and Methods

### 4.1. Location of Soil Material

There were three substrates used in this study, namely NS, based on soil from landfill near Novi Sad (N 45°18′, E 19°50′, altitude 75 m a.s.l., Serbia); BG, based on soil from landfill near Vinča (in the vicinity of Belgrade) (N 44°47′, E 20°36′, altitude 99 m a.s.l., Serbia); and control, based on soil from experimental estate of the Institute of Lowland Forestry and Environment (ILFE) of the University of Novi Sad in Kać (in the vicinity of Novi Sad) (N 45°17′, E 19°53′, 78 m a.s.l., Serbia).

### 4.2. Granulometric Composition and Chemical Properties of Substrates

Particle size distribution (%) was determined by the international B-pipette method, which involved preparation in sodium pyrophosphate. Soil textural classes were determined based on particle size distribution using the Atterberg classification. The CaCO_3_ percentage (%) was measured volumetrically using a Scheibler calcimeter (Royal Eijkelkamp, Giesbeek, The Netherlands). pH in H_2_O was determined with an electrometric method with a combined electrode on a Radiometer pH meter (InoLab^®^ pH/ION/Cond 750, WTW, Wellheim, Germany). The content of humus was measured by the Tyurin method using the Simakov modification. Total nitrogen was measured using to the Kjeldahl method, and easily accessible phosphorus and potassium were measured using to the AL method. All analyses were performed in the Laboratory of Soil Science at the Institute of Lowland Forestry and Environment in Novi Sad, using the methodology under quality assurance and control described by [44] and the ICP Forests manual for soil sampling and analysis [45].

### 4.3. Heavy Metal Content Analysis

The substrate and shoot samples were dried in oven at 105 °C, then grounded and resolved in aqua regia to prepare samples for the extraction of heavy metals. The digestion was performed in a microwave digestion platform (Milestone D series, Bergamo, Italy). The resulting extract was filtered and filled up to 50 mL. The content of heavy metals in the obtained extracts, in aqua regia, was measured by ICP-OES spectrometer VISTA-PRO (Varian Australia Pty. Ltd., Melbourne, VIC, Australia). The radiofrequency power was set to 1100 W, ensuring efficient plasma generation, while the plasma gas flow rate was maintained at 15.0 L min^−1^ for stable plasma operation. An auxiliary gas flow rate of 1.50 L min^−1^ was employed to support plasma stability. Following the methodology of [37], the measurements were performed in three replicates. One sample per substrate and three plant shoot samples per clone were measured. The values obtained in this way were compared with the maximum limit value (MLV) and remediation value (RV) of heavy metals in soil [30].

### 4.4. Plant Material

Seven clones were evaluated in the pot trials, including three eastern cottonwood clones (*Populus deltoides* Bartr.): PE19/66, S1-8, and 135/81; two euramerican poplar clones (*Populus × euramericana* Dode (Guinier)): I-214 and Pannonia; and two white willow clones (*Salix alba* L.): 107/65-9 and 380. The clones were obtained from the ILFE gene bank. Pot trials were established in April 2023, with three replicates per substrate per clone. Two one-year hardwood cuttings per clone were planted in 3 L pots, and the plants were cultivated under semi-controlled greenhouse conditions at ILFE. The irrigation regime was maintained at optimal levels, with watering performed twice a week to prevent water stress. The duration of the growth period spanned 90 days, beginning with cuttings establishment and concluding at the designated sampling time. The plants were cultivated under controlled conditions, with a photoperiod of 16 h of light and 8 h of darkness, an average temperature of 21 ± 2 °C, and relative humidity maintained at 60–70%. The sampling was conducted at the end of the growing season, specifically 120 days after planting.

Also, in order to study the heavy metal transfer rate from soil to the shoot, i.e., to evaluate the plants’ phytoextraction ability, the bioavailability factor was calculated by the following formula, according to [46]:

BF = C_shoot_/C_soil_, (1)

where BF stands for bioavailability factor, C_shoot_ for content of heavy metal in shoot [mg/kg DW], and C_soil_ for the content of heavy metal in substrate [mg/kg DW].

### 4.5. Statistical Analysis

The data analysis included two-way factorial analysis of variance, where Substrate and Clone were the main effects, followed by Tukey’s honestly significant difference (HSD) post hoc test for multiple comparisons of treatments, following a two-way analysis of variance (ANOVA) that indicated significant differences (*p* < 0.01). The analysis focused on identifying significant differences (i) between treatments, (ii) between clones, and, (iii) in the interaction, between clones and treatments at the level α = 0.05. The relationship between treatments at the level of interaction was also examined by principal component analysis based on the first two unrotated principal components. Furthermore, principal component analysis was used for studying the relations between the examined parameters as well. The parameters were grouped based on their highest factor loadings with some of the first four principal components. These principal components were rotated by the Varimax method in order to maximize variance of factor loadings within the principal components. All statistical analyses were carried out with the STATISTICA 14.0.0 software package (TIBCO Software Inc., 2020, Palo Alto, CA, USA) [47].

## 5. Conclusions

This study evaluated the phytoextraction capabilities of willow and poplar clones at the base of pot trial on two landfill soil-based substrates based on their heavy metal accumulation patterns. Willow clones 107/65-9 and 380 were found to act as generalists, showing considerable accumulation of numerous heavy metals analyzed. These clones are recommended for sites with various contaminants, especially where water availability is high. It seems that the bioavailability factor of heavy metals is lower on substrate with higher clay + silt content and higher soil content of heavy metals. Certain poplar clones, like I-214 and Pannonia, acted as specialists, excelling in accumulation of some specific metals (Cu, Mn, and Zn), which supported their suitability for targeted phytoremediation at sites contaminated with metals for which they showed affinity. This study underlines the importance of site-specific selection of poplar and willow clones for establishing plantations for phytoremediation on landfills based on heavy metal contamination patterns and growing conditions. Further field trials are necessary to refine these findings and better understand how to optimize phytoremediation strategies for various contaminated environments.

## Figures and Tables

**Table 1:** Granulometric composition and chemical properties of experimental substrates.

Granulometric Composition							
Substrate	Coarse Sand (%)	Fine Sand (%)	Silt (%)	Clay (%)	Total Sand (%)	Total Clay (%)	Soil Texture
Control (Kać)	42.09	47.03	7.68	3.2	89.12	10.88	Sand
Belgrade landfill	4.28	35.68	28.76	31.28	39.96	60.04	Clay loam
Novi Sad landfill	22.08	41.76	19.24	16.92	63.84	36.16	Sandy loam
**Chemical Properties**							
	**CaCO_3_** **(%)**	**pH** **(in H_2_O)**	**Humus** **(%)**	**N** **(%)**	**P_2_O_5_** **(mg/100 g)**	**K_2_O** **(mg/100 g)**	
Control (Kać)	11.12	8.09	1.65	0.016	3.85	2.92	
Belgrade landfill	6.82	7.95	2.14	0.133	12.66	10.28	
Novi Sad landfill	13.06	8.62	2.71	0.076	8.38	6.70	

**Table 2:** Heavy metal content of experimental substrates with their maximum limit value (MLV) and remediation value (RV).

Substrate	Heavy Metal Content (mg/kg)										
	As	Ba	Cd	Cr	Cu	Fe	Hg	Mn	Ni	Pb	Zn
Control (Kać)	n.d. ^(1)^	16.30	n.d.	50.77	25.61	8343.87	n.d.	163.20	15.91	14.50	28.03
Novi Sad landfill	n.d.	21.10	1.20 * ^(2)^	64.60	33.10	10,777.50	1.50	210.80	21.60	23.80	48.00
Belgrade landfill	3.69	109.00	1.83 *	90.98	37.42 *	33,425.00	n.d.	685.20	77.68 *	46.60	65.70
MLV	29	160	0.8	100	36	/	0.3	/	35	85	140
RV	55	625	12	380	190	/	10	/	210	530	720

^(1)^ n.d. stands for not detected. ^(2)^ * stands for values that exceed MLV of the heavy metal in soil.

**Table 3:** F-test based on the two-way factorial analysis of variance for the content of the examined heavy metals in poplar and willow clones.

Source of Variation	Cadmium		Crome		Cupper		Iron		Manganese		Nickel		Lead		Zinc	
	F ^(^*^)^	*p*	F	*p*	F	*p*	F	*p*	F	*p*	F	*p*	F	*p*	F	*p*
Substrate (A)	75.90	0.000	77.53	0.000	4.77	0.014	60.17	0.000	132.36	0.000	64.64	0.000	76.24	0.000	12.13	0.000
Clone (B)	99.80	0.000	21.71	0.000	13.79	0.000	22.25	0.000	13.20	0.000	25.85	0.000	9.30	0.000	51.63	0.000
Interaction A × B	9.91	0.000	12.86	0.000	1.18	0.326	10.10	0.000	3.75	0.001	3.84	0.001	9.19	0.000	2.61	0.010

^(^*^)^ F stands for F-value and *p* stands for *p*-value.

**Table 4:** Tukey’s HSD test for heavy metal content in shoot tissue of the examined clones on landfill soil-based substrates.

Treatments		Content in Shoot Tissue (mg/kg DW)															
Substrate ^(a)^	Clone ^(c)^	Cadmium		Crome		Copper		Iron		Manganese		Nickel		Lead		Zinc	
**Means of Substrates**																	
BG		1.93	^a (b)^	5.63	^a^	52.14	^a^	666.37	^a^	85.28	^a^	7.22	^a^	3.26	^a^	129.17	^a^
NS		1.51	^b^	3.90	^b^	48.39	^b^	425.79	^b^	57.47	^b^	4.75	^b^	2.76	^b^	110.90	^b^
Control		1.50	^b^	3.68	^b^	47.78	^b^	232.73	^c^	47.01	^c^	4.81	^b^	1.91	^c^	122.49	^a^
**Means of Clones**																	
	*107/65-9*	2.40	^a^	6.03	^a^	52.59	^ab^	827.33	^a^	44.25	^c^	6.58	^a^	3.24	^a^	115.75	^cd^
	*380*	1.98	^b^	4.88	^b^	43.40	^cd^	574.61	^b^	62.48	^b^	4.29	^bc^	2.94	^ab^	138.55	^b^
	I-214	1.44	^d^	4.05	^c^	56.05	^a^	363.55	^c^	62.26	^b^	3.99	^c^	2.28	^c^	111.84	^d^
	Pannonia	1.35	^d^	4.16	^bc^	56.60	^a^	326.70	^c^	71.19	^ab^	5.26	^b^	2.41	^bc^	115.28	^cd^
	PE19/66	1.14	^e^	4.08	^bc^	41.12	^d^	332.59	^c^	74.43	^a^	4.76	^bc^	2.32	^c^	69.11	^e^
	S1-8	1.68	^c^	4.10	^bc^	48.62	^bc^	350.40	^c^	62.27	^b^	7.36	^a^	2.69	^bc^	163.00	^a^
	135/81	1.49	^d^	3.40	^c^	48.18	^bcd^	278.24	^c^	64.18	^ab^	6.96	^a^	2.52	^bc^	130.64	^bc^
**Means at the Level of Interaction Substrate × Clone**																	
BG	*107/65-9*	3.04	^a^	9.16	^a^	54.89	^abc^	1498.81	^a^	75.79	^abcde^	9.51	^a^	4.79	^a^	138.90	^abcd^
	*380*	2.25	^bc^	7.56	^a^	44.65	^bcd^	1110.83	^a^	90.95	^ab^	6.79	^bc^	4.42	^a^	145.23	^abc^
	I-214	1.65	^defghi^	4.69	^bcd^	59.36	^ab^	427.72	^bc^	83.20	^abc^	4.90	^cdefgh^	2.64	^bcdef^	112.30	^cdef^
	Pannonia	1.75	^defg^	4.71	^bcd^	57.19	^ab^	428.11	^bc^	99.75	^a^	6.91	^bc^	2.82	^bcde^	140.20	^abcd^
	PE19/66	1.39	^fghijkl^	5.06	^b^	46.10	^abcd^	458.17	^bc^	100.05	^a^	6.47	^bcd^	2.88	^bcde^	67.29	^hi^
	S1-8	1.95	^cde^	3.94	^bcde^	53.17	^abc^	324.44	^bc^	65.56	^cdef^	7.94	^ab^	2.71	^bcdef^	168.92	^a^
	135/81	1.47	^fghijk^	4.32	^bcde^	49.65	^abcd^	416.54	^bc^	81.67	^abcd^	8.00	^ab^	2.59	^cdef^	131.35	^abcde^
NS	*107/65-9*	1.76	^defg^	4.69	^bcd^	53.57	^abc^	698.02	^b^	34.23	^gh^	4.30	^defgh^	3.14	^bc^	87.78	^fghi^
	*380*	1.98	^cd^	4.36	^bcde^	40.76	^cd^	517.08	^bc^	54.94	^efg^	3.28	^h^	2.55	^cdef^	136.31	^abcde^
	I-214	1.37	^ghijkl^	3.32	^cde^	57.33	^ab^	342.46	^bc^	57.83	^defg^	3.20	^h^	2.28	^cdef^	104.35	^defgh^
	Pannonia	1.19	^jkl^	3.16	^de^	52.10	^abcd^	298.94	^bc^	64.82	^cdef^	3.93	^efgh^	2.44	^cdef^	99.17	^efghi^
	PE19/66	1.03	^l^	3.63	^bcde^	37.46	^d^	337.23	^bc^	66.56	^bcdef^	4.18	^defgh^	2.12	^cdef^	65.70	^i^
	S1-8	1.57	^efghij^	4.99	^bc^	48.41	^abcd^	530.50	^bc^	69.64	^bcdef^	7.78	^ab^	3.77	^ab^	151.53	^ab^
	135/81	1.70	^defgh^	3.14	^de^	49.12	^abcd^	256.29	^c^	54.29	^efg^	6.57	^bcd^	3.03	^bcd^	131.43	^abcde^
Control	*107/65-9*	2.41	^b^	4.23	^bcde^	49.30	^abcd^	285.15	^c^	22.72	^h^	5.92	^bcdefg^	1.80	^ef^	120.57	^bcdef^
	*380*	1.78	^def^	3.25	^de^	44.43	^bcd^	215.60	^c^	46.79	^fgh^	3.17	^h^	2.12	^cdef^	135.21	^abcde^
	I-214	1.28	^ijkl^	4.15	^bcde^	51.47	^abcd^	320.47	^bc^	45.77	^fgh^	3.88	^fgh^	1.93	^def^	118.86	^bcdef^
	Pannonia	1.12	^kl^	4.61	^bcd^	60.51	^a^	253.07	^c^	49.00	^fg^	4.95	^cdefgh^	1.97	^def^	106.46	^defg^
	PE19/66	0.99	^l^	3.53	^bcde^	39.81	^cd^	202.35	^c^	56.66	^efg^	3.64	^gh^	1.95	^def^	74.34	^ghi^
	S1-8	1.53	^fghij^	3.37	^bcde^	44.28	^bcd^	196.27	^c^	51.60	^efg^	6.34	^bcde^	1.61	^f^	168.56	^a^
	135/81	1.30	^hijkl^	2.75	^e^	45.76	^abcd^	161.89	^c^	56.59	^efg^	6.31	^bcdef^	1.93	^def^	129.15	^bcde^

^(a)^ Substrate labels: BG—landfill soil from Vinca (Belgrade); NS—landfill soil from Novi Sad, Control.—soil from ILFE experimental estate in Kać (Novi Sad). ^(b)^ Values with the same letter are not significantly different according to Tukey’s HSD test for α = 0.05. ^(c)^ Labels of willow clones are in italic, and labels of poplar clones are in normal font.

**Table 5:** Factor loadings of original parameters with the first four principal components rotated by Varimax method.

Shoot Content	Rotated Principal Component ^(^*^)^			
	1	2	3	4
Cadmium	0.785	0.489	−0.200	0.056
Chromium	0.927	0.093	0.237	0.104
Copper	0.090	0.076	0.037	0.992
Iron	0.969	0.059	0.167	0.040
Manganese	0.207	0.005	0.942	0.028
Nickel	0.383	0.654	0.479	0.093
Lead	0.850	0.161	0.365	0.051
Zinc	0.079	0.948	−0.041	0.052
Eigenvalue Contribution to the total variance	3.342	1.610	1.376	1.015
	0.418	0.201	0.172	0.127

^(^*^)^ Underlined factor loading of an original variable is its greatest among rotated principal components.

**Table 6:** Bioavailability factor (BF) for heavy metals in the examined clones on landfill soil-based substrates.

Substrate ^(a)^	Clone ^(b)^	Cadmium	Chromium	Copper	Iron	Manganese	Nickel	Lead	Zinc
BG	*107/65-9*	1.660	0.101	1.467	0.045	0.111	0.122	0.103	2.114
	*380*	1.232	0.083	1.193	0.033	0.133	0.087	0.095	2.211
	I-214	0.904	0.052	1.586	0.013	0.121	0.063	0.057	1.709
	Pannonia	0.954	0.052	1.528	0.013	0.146	0.089	0.061	2.134
	PE19/66	0.760	0.056	1.232	0.014	0.146	0.083	0.062	1.024
	S1-8	1.065	0.043	1.421	0.010	0.096	0.102	0.058	2.571
	135/81	0.801	0.047	1.327	0.012	0.119	0.103	0.056	1.999
NS	*107/65-9*	1.466	0.073	1.618	0.065	0.162	0.199	0.132	1.829
	*380*	1.647	0.067	1.231	0.048	0.261	0.152	0.107	2.840
	I-214	1.144	0.051	1.732	0.032	0.274	0.148	0.096	2.174
	Pannonia	0.990	0.049	1.574	0.028	0.307	0.182	0.102	2.066
	PE19/66	0.862	0.056	1.132	0.031	0.316	0.193	0.089	1.369
	S1-8	1.309	0.077	1.462	0.049	0.330	0.360	0.158	3.157
	135/81	1.416	0.049	1.484	0.024	0.258	0.304	0.127	2.738
Control	*107/65-9*	-	0.083	1.925	0.034	0.139	0.372	0.124	4.302
	*380*	-	0.064	1.735	0.026	0.287	0.200	0.146	4.824
	I-214	-	0.082	2.010	0.038	0.280	0.244	0.133	4.240
	Pannonia	-	0.091	2.363	0.030	0.300	0.311	0.136	3.798
	PE19/66	-	0.070	1.555	0.024	0.347	0.229	0.135	2.652
	S1-8	-	0.066	1.729	0.024	0.316	0.399	0.111	6.014
	135/81	-	0.054	1.787	0.019	0.347	0.397	0.133	4.607

^(a)^ Substrate labels: BG—landfill soil from Belgrade; NS—landfill soil from Novi Sad; Control—soil from ILFE estate in Kać (Novi Sad). ^(b)^ Labels of willow clones are in italic, and labels of poplar clones are in normal font.

## Data Availability

All data are included in the manuscript.
